# A Hypothetical Experiment: Evaluating the Real-world Impact of Artificial Intelligence Use in Academic Writing

**DOI:** 10.31662/jmaj.2024-0411

**Published:** 2025-03-07

**Authors:** Shigeki Matsubara

**Affiliations:** 1 Department of Obstetrics and Gynecology, Jichi Medical University, Tochigi, Japan; 2 Department of Obstetrics and Gynecology, Koga Red Cross Hospital, Koga, Japan; 3 Medical Examination Center, Ibaraki Western Medical Center, Chikusei, Japan

**Keywords:** artificial intelligence, experiment, journal, regulation, writing

## Dear Editors,

I thank Professor Mori ^(1)^ for responding to my Letter ^(2)^. Our thoughts on artificial intelligence (AI) use in academic writing fundamentally agree: that a “standard guideline” is necessary. However, the lack of “real-world” data prevents its establishment. To tentatively settle our discussion and offer a possible path forward, I propose a simple hypothetical experiment.

A certain journal should “declare”: (1) “AI use is freely permissible under the condition that the authors fully explain how AI was used,” and (2) “It is completely prohibited.” Employ strategy (1) for 202X and (2) for 202X+1. Then, compare all the data between the (1)- and (2)-eras, emphasizing i) papers’ readability and significance, ii) citation numbers (to be determined later), and iii) overall journal reputation. During the 1)-era, how AI was used, including its degree, should also be determined. Please put aside some difficulties: whether AI use will be honestly declared and how to evaluate readability, significance, and reputation.

AI use has both merits and demerits, which creates complexity. Those weighing demerits over merits advocate for strict regulation, while their counterparts support a less strict one ^(3)^. Importantly, these merits and demerits are discussed based on a hypothetical scenario. There are no actual experiments directly demonstrating them in the “real-world” context of journals. This is an overstatement; it is akin to experimenting on non-human animals rather than humans to understand humans, which complicates the argument further.

In the initial phase of studies, experiments often focus on related aspects rather than the primary target, with the obtained data being extrapolated. However, we should step into a new phase. This hypothetical experiment may not solve the problem all at once; however, if one wishes to understand the “real-world effect” of AI use, one must experiment within the journals’ “real world.” Indeed, comparisons before and after journals transitioned to fully open access objectively showed its merits and demerits ^(4)^.

The problems are: (1) Which journal will undertake this? (2) Can we design a study that yields meaningful analysis? (3) Can the journal help authors and readers understand the experiment’s purpose without confusion? Regarding (1), I believe that a relatively smaller circulation journal could be the first to conduct a pilot study. The journal community, including the Japan Medical Association Journal, may provide necessary support.

Such an experiment cannot answer the most important question: whether AI use in writing will influence future human writing ability and cognitive function ^(5)^. Only time will tell.

## Article Information

### Conflicts of Interest

None

### Author Contributions

Shigeki Matsubara: Identification of the significance and manuscript writing.

### Approval by Institutional Review Board (IRB)

Not applicable.

### Data Availability

Data sharing is not applicable to this article, as no new data were created or analyzed in this study.

### Patient Anonymity

Not applicable.

### Informed Consent

Not applicable.
